# The Impact of Social Determinants of Health on Treatment Received in Patients with Stage I Lung Cancer in Ontario: A Population-Based Analysis

**DOI:** 10.3390/curroncol32120713

**Published:** 2025-12-18

**Authors:** Nader M. Hanna, Saad Shakeel, Gileh-Gol Akhtar-Danesh, Christian Finley, Noori Akhtar-Danesh

**Affiliations:** 1 Department of Surgery, McMaster University, Hamilton, ON L8N 4A6, Canada; nader.hanna@medportal.ca (N.M.H.); finleyc@mcmaster.ca (C.F.); 2 Department of Surgery, University of Western Ontario, London, ON N6A 5A5, Canada; sshakee4@uwo.ca; 3 Department of Surgery, University of Manitoba, Winnipeg, MB R3A 1R9, Canada; gileh-gol.akhtar-danesh@medportal.ca; 4 School of Nursing, McMaster University, Hamilton, ON L8S 4K1, Canada

**Keywords:** lung cancer, social determinants of health, access to care, population-level study

## Abstract

Surgical resection is recommended for operable stage I non-small-cell lung cancer (NSCLC), while radiotherapy reserved for inoperable patients. Very comorbid patients may receive no treatment at all. Social determinants of health (SDOHs) may influence access to these treatments. We examined how SDOHs affect treatment modality among these patients using a population-based retrospective cohort study using ICES data including adults with stage I NSCLC diagnosed between 2007 and 2023. Multivariable logistic regression assessed associations between SDOH and treatment received. Of 19,179 patients, 54.4% received only surgery, 15.8% received only radiotherapy, 27.5% received no treatment, and 2.3% received surgery and radiotherapy. Surgery was less likely in patients aged >80 versus <50 (OR 0.07, *p* < 0.001), patients with frailty (OR 0.38, *p* < 0.001), patients with ≥5 comorbidities (OR 0.21, *p* < 0.001), or those who were not rostered with a family physician (OR 0.59, *p* < 0.001). Recent immigrants were more likely to undergo surgery (OR 1.23, *p* = 0.035), as well as those in the highest neighbourhood income quintile (OR 1.45, *p* < 0.001). Surgery was less likely for those living 50–100 km from a cancer centre (OR 0.85, *p* = 0.004). Radiotherapy was more likely in patients aged >80 (OR 9.86, *p* < 0.001), those with ≥5 comorbidities (OR 2.23, *p* < 0.001), or those in the lowest household income quintile (OR 1.27, *p* = 0.009). Recent immigrants were less likely to receive radiotherapy (OR 0.69, *p* = 0.005). SDOHs independently influence treatment type for stage I NSCLC.

## Simple Summary

Lung cancer is the leading cause of cancer death in Canada, but when caught early at stage I, it can often be cured—usually with surgery. However, social determinants of health, such as income, access to a family doctor, immigration status, and where a person lives, can shape the care they receive. We studied over 19,000 Ontarians with stage I lung cancer to see how these factors influenced treatment. Just over half had surgery, while others received radiation or no treatment. Patients without a family physician, those living farther from a cancer centre, and those in lower-income neighbourhoods were less likely to receive surgery. Older adults and patients with many health issues were also less likely to receive surgery and more likely to receive radiation. Recent immigrants were more likely to undergo surgery than long-term residents. Overall, social and structural factors strongly influence who receives guideline-recommended lung cancer treatment in Ontario.

## 1. Introduction

Despite advances in early detection and treatment, significant disparities persist in the management of stage I non-small-cell lung cancer (NSCLC) in Ontario, with social determinants of health (SDOHs) playing a critical role in determining access to guideline-recommended care. According to clinical guidelines [1], surgical resection is the preferred curative treatment for operable stage I NSCLC (consistently across AJCC editions), offering superior long-term survival compared to stereotactic body radiotherapy (SBRT). Further still, patients who are extremely elderly or comorbid might not receive any treatment because the risks of receiving treatment may outweigh the benefits. Mounting evidence suggests that patients from socioeconomically disadvantaged backgrounds, those without a regular primary care provider, recent immigrants, and individuals living far from cancer centres are less likely to receive surgical treatment and more likely to be treated with radiotherapy [2,3,4,5].

One key driver of this disparity is access to lung cancer screening. Organized screening programmes in Ontario, initiated in 2021, rely heavily on primary care provider referrals. Individuals without a family physician are less likely to be referred for screening or followed appropriately after abnormal imaging. This delay can result in missed opportunities for timely diagnosis, surgical consultation, and operative management. For most of the study period (2007–2020), stage I lung cancers were identified through opportunistic imaging or investigation of symptoms, with access to timely diagnostic assessment and specialist referral mediated largely by primary-care engagement. Furthermore, surgery for NSCLC is centralized in thoracic surgical centres [6], typically located in urban areas. Patients residing in rural or remote regions face logistical and financial barriers to accessing surgical care [7], making outpatient-based radiotherapy a more accessible, albeit less optimal alternative [4,5].

In Ontario, population-based studies using linked administrative and cancer registry data have demonstrated that patients living in more deprived or marginalized neighbourhoods are more likely to present with advanced-stage cancer and have poorer survival [2,8]. Building on this work, our study focuses specifically on how social determinants influence receipt of surgery, radiotherapy, or no treatment among patients with stage I NSCLC.

## 2. Materials and Methods

### 2.1. Study Design and Cohort

We conducted a population-based retrospective cohort study using routinely collected administrative healthcare databases and registries housed at the Institute for Clinical Evaluative Sciences (ICES). These datasets were linked using unique encoded identifiers and analyzed at ICES. ICES is an independent, non-profit research institute whose legal status under Ontario’s health information privacy law allows it to collect and analyze health care and demographic data, without consent, for health system evaluation and improvement. Ontario has over 16 million residents, making it the most populous Canadian province. We used the Ontario Cancer Registry (OCR) to identify adult patients diagnosed with lung cancer between January 2007 and December 2023 using the following ICD-O codes: C34.0 (malignant neoplasm of main bronchus), C34.1 (malignant neoplasm of upper lobe, bronchus or lung), C34.2 (malignant neoplasm of middle lobe, bronchus or lung), C34.3 (malignant neoplasm of lower lobe, bronchus or lung), C34.8 (overlapping malignant lesion of bronchus or lung), and C34.9 (malignant neoplasm of bronchus or lung, unspecified). We selected stage I patients (using the *beststage* variable that prioritizes pathological stage where available) and identified those who underwent either radiotherapy (via the cancer Activity Level Reporting database (ALR)) or surgery (via the Canadian Classification for Health Interventions (CCI)). The ICES ‘beststage’ algorithm synthesizes stage information from multiple data sources and assigns a single TNM stage for each patient, prioritizing pathological over clinical staging when both are available. In our cohort, pathological staging was available for all patients who underwent surgical resection, whereas patients treated with radiotherapy alone or had no treatment were staged on clinical grounds. Patients who received chemotherapy were excluded as this may reflect misclassified higher-stage disease, adjuvant therapy for high-risk features, or treatment of another malignancy. To focus on treatment decisions for a single incident lung cancer, we excluded individuals with another invasive cancer diagnosed within five years before or after the index stage I lung cancer. This conservative approach reduces the risk that treatments directed at a different primary malignancy would be misattributed to the index lung cancer. The Hamilton Integrated Research Ethics Board reviewed and approved the research proposal for this study (HiREB #17274).

### 2.2. Data Sources

The OCR captures demographic, diagnostic, and death information on over 98% of incident cancers [9]. This was linked to other databases to obtain outcomes and covariate data. The ALR provides information on dose, timing, and dates of radiotherapy. From the CCI we obtained surgical information. Demographic data and covariates were sourced from the Registered Persons Database (RPDB), the Ontario Health Insurance Plan (OHIP), the Same Day Surgery (SDS) database, the Discharge Abstract Database (DAD), the Local Health Integration Network (LHIN), Postal Code Conversion File (PCCF), the Ontario Marginalization Database (ONMARG), the National Ambulatory Care Reporting System (NACRS), and the IRCC Permanent Residents database (CIC).

### 2.3. Covariates and Outcomes

The primary outcome was type of treatment received (radiotherapy or surgery). Demographic data including age, sex, comorbidity index (gathered from 6 to 30 months before the diagnosis date using OHIP and DAD, and categorized based on the Johns Hopkins ACG System [10]), frailty index (also calculated based on the Johns Hopkins ACG System), and year of diagnosis were all categorized. The 14 LHINs in Ontario were grouped into four geographic regions: Central, Southwest, East, and North. SDOHs included household income, neighbourhood income, family physician status, immigration status, and distance to the nearest regional cancer centre. Those who immigrated to Canada ≤5 years before their diagnosis were classified as new or recent immigrants.

### 2.4. Statistical Analysis

Descriptive statistics were used to describe the cohort. We used the Chi-Square test to examine the association between treatment type and each variable. Subsequently, we created a radiotherapy group and a surgery group and performed a multiple logistic regression analysis to identify factors associated with receipt of each type of treatment. For descriptive analyses, we retained individuals with missing covariate data. Multivariable logistic regression models were estimated using complete-case analysis, excluding patients with missing values on any model covariates. We assessed multicollinearity and found no evidence of this among included covariates. Several clinically plausible interaction terms were explored but did not materially change the main-effect estimates and were not retained in final models. Statistical analyses were conducted using Stata/MP 15.1 (Stata Corporation, College Station, TX, USA).

## 3. Results

We identified 20,393 patients diagnosed with stage I lung cancer between January 2007 and December 2023. After excluding those who received chemotherapy, 19,179 remained, of which 5275 received no treatment. The remaining received surgery alone (10,432), radiotherapy alone (3026), or surgery and radiotherapy (446). Table 1 presents the baseline characteristics of the cohort. Females comprised 56.9% of the entire cohort, and most patients were aged 70 years and above (59.4%). Most patients (50.5%) had at least one comorbidity based on the CCI and 11.4% were frail. In total, 27.5% of patients received no treatment, 54.4% had surgery alone, 15.8% received radiation alone, and 2.3% received both surgery and radiation. Patients who received no treatment were, on average, older and more frail, with a higher burden of comorbidities and less consistent attachment to a family physician, compared with those who underwent surgery or radiotherapy.

Table 2 depicts the results of the logistic regression analysis for the association of patient demographics and SDOHs on receiving surgery. Females were significantly more likely to undergo surgery (OR = 1.17, *p*-value < 0.001). The likelihood of surgery significantly decreased with advancing age (e.g., OR 0.07, *p*-value < 0.001 for >80 years compared to <50 years), frailty (OR = 0.38, *p*-value < 0.001), and increasing number of comorbidities (OR = 0.50, *p*-value < 0.001 for 1–2 comorbidities; OR = 0.31, *p*-value < 0.001 for 3–4 comorbidities; OR = 0.21, *p*-value < 0.001 for 5 or more comorbidities, respectively). The likelihood of undergoing surgery significantly decreased over time, at the lowest for years 2020–2023 compared to 2007–2009 (OR = 0.33, *p*-value < 0.001), and those in the Eastern region were the least likely to receive surgery (OR = 0.36, *p*-value < 0.001 compared to those in the Central region).

Patients in income quintiles 2–5 were significantly more likely to receive surgery than those in the lowest income quintile (ORs 1.32, 1.35, 1.35, 1.45, respectively, *p*-value < 0.001), whereas those in the lowest household income quintile were less likely to receive surgery compared to the highest quintile (OR = 0.69, *p*-value < 0.001). Recent immigrants were more likely to receive surgery than non-immigrants (OR = 1.23, *p*-value = 0.035). Patients who lived 50–100 km from the nearest regional cancer centre had significantly lower chances of having surgery compared to those who lived less than 50 km away (OR = 0.84, *p*-value = 0.004), but those who lived >100 km away were just as likely to receive surgery as those living <50 km away (OR 0.93, *p*-value = 0.449). Compared to those who were fully rostered with a family physician, those who were only virtually rostered and those not rostered had a lower chance of receiving surgery (OR 0.85, *p*-value = 0.009; OR 0.59, *p*-value = 0.001, respectively).

Table 3 illustrates the demographic and SDOH factors associated with receiving radiotherapy treatment. Females were significantly less likely to receive radiotherapy compared to males (OR = 0.85, *p* = 0.001). The likelihood of receiving radiotherapy significantly increased with advancing age, highest for patients aged ≥80 years compared to those <50 (OR = 9.86, *p*-value < 0.001). Increasing number of comorbidities was associated with a higher chance of receiving radiotherapy (e.g., OR = 2.24, *p* < 0.001 for those with >5 comorbidities compared to those without comorbidities). The likelihood of receiving radiotherapy significantly decreased over time between the periods of 2015–2019 (OR = 0.81, *p*-value < 0.001) and 2020–2023 (OR = 0.24, *p*-value < 0.001), compared to 2007–2009. Those living in the Eastern region had the highest likelihood of receiving radiotherapy (OR = 2.11, *p*-value < 0.001 compared to those in the Central region).

Those in household quintiles 4 and 5 had the highest chance of receiving radiotherapy compared to the lowest quintile (OR = 1.21, *p*-value = 0.05; OR = 1.27, *p*-value = 0.009, respectively). Recent immigrants had a significantly lower likelihood of receiving radiotherapy compared to non-immigrants (OR = 0.69, *p*-value = 0.005). Neighbourhood income, frailty, distance from the regional cancer centre and access to a family physician were not significant contributors to the model, and hence were excluded from the multivariable analysis.

## 4. Discussion

In this population-based study, we found significant differences in the type of treatment received for stage I lung cancer based on demographics and SDOHs. Those who received surgery were more likely to be younger, female, less comorbid, and not frail, lived in the Central region, were fully rostered with a family physician, lived in a high-income neighbourhood or had a high-income household, and were recent immigrants. This is in line with other research investigating the impact of SDOHs on surgery for lung cancer in Denmark [11] and in the USA [12], and on guideline-concordant treatment of esophageal cancer [13]. The finding that more than one-quarter of patients with stage I NSCLC received no cancer-directed treatment is concerning. While some of these patients may have been appropriately managed with best supportive care because of extreme frailty, multiple comorbidities, or patient preference, others may represent potentially avoidable undertreatment. A deeper understanding of patient, clinician, and system-level factors contributing to non-treatment is needed to ensure that curative-intent options are equitably offered and accessible.

In the univariable analysis, we observed an increase in the number of patients receiving surgery and a decrease in the number of patients receiving radiotherapy in a temporal fashion during the study period 2007–2023. This is likely a real-world reflection of the surgical and oncology communities embracing clinical guidelines. In the multivariable analysis, however, we found that the likelihood of receiving surgery and the likelihood of receiving radiotherapy reduced as time went on (compared to the reference period of 2007–2009). We chose to group the years in this manner to reflect recent policy change (the process of regionalisation of thoracic surgery centres from 2007 to 2010) [6,14] and the COVID-19 pandemic (2019 onwards). A recent global systematic review [15] investigating the impact of the pandemic on receipt of treatment demonstrated a 15% reduction in radiotherapy services and a 29% reduction in surgical procedures for cancer during the pandemic compared to the pre-pandemic period, in line with our own findings. These adjusted temporal trends likely reflect a combination of evolving referral patterns, increased availability and acceptance of SBRT, regionalization of thoracic surgery, and system-level disruptions caused by the COVID-19 pandemic, which led to delays and reductions in cancer surgery in many LHINs.

Studies comparing radiotherapy with surgery for early-stage lung cancer draw conflicting conclusions [16,17,18,19,20]. However, surgical guidelines [1] advocate for surgical resection as first-line treatment in those deemed operable, with radiotherapy being reserved for non-surgical candidates or those who decline surgery. Cited benefits of surgery over radiotherapy include definitive treatment (by physically removing the tumour), confirming the diagnosis of early-stage NSCLC, and obtaining adequate lymph node samples to more accurately stage the patient. In our analyses, those who were younger, less comorbid, and not considered frail were more likely to receive surgery compared to radiotherapy, which is concordant with guidelines. Importantly, we identified key SDOHs that impacted the type of treatment received despite controlling for age, comorbidity, and frailty.

In the present analysis, patients not rostered with a family physician were less likely to receive surgery. One possible explanation is that these patients may not have access to the same pathways that patients with a family physician do. For example, they may be less likely to be referred to a Lung Diagnostic Assessment Pathway (LDAP) that is often surgeon-led. NSCLC is generally asymptomatic in the early stages and is often an incidental finding during the workup of unrelated symptoms. Whilst purely speculative, it is possible that patients without a family physician seek treatment in either the emergency department or an urgent care centre and are diagnosed by providers less familiar with lung cancer management. Thus, it is also possible that these patients may not be referred to the appropriate specialist through pre-existing channels. Previous studies demonstrate that enrollment in an LDAP programme leads to enhanced staging completeness and reduced staging time, and is correlated with better survival rates [21,22,23]. Therefore, access to appropriate care remains an important yet understudied element of patients without family physicians.

Recent immigration was associated with a higher likelihood of receiving surgery for stage I lung cancer. Previous studies report conflicting findings regarding the effect of immigration on the diagnosis, staging, and treatment of lung cancer [8,24]. However, one recent population-level study [3] found that survival for lung cancer was better in Ontario immigrants compared to long-term residents. This may be explained by the “healthy immigrant effect”, whereby immigrants are more likely to be younger, healthier, and better educated and have higher health literacy compared to non-immigrants. Additionally, immigrants are less likely to smoke compared to Ontario natives [25], which may impact their care and referral patterns. Despite being more likely to live in lower-income neighbourhoods [26], our analysis demonstrated that immigrants had a higher likelihood of receiving surgery for lung cancer when accounting for SES.

Patients in higher-income neighbourhoods were more likely to receive surgery than those in lower-income neighbourhoods. This is not a new finding in Ontario [24], but the causes for this inequality are still unclear. One possible explanation is lower health literacy within lower-income neighbourhoods [27], and a resultant inability to advocate for oneself regarding the most appropriate treatment. Another potential reason is that the postoperative course after surgery for lung cancer can be quite burdensome—patients may need frequent follow up and repeat prescriptions, and some require home care for medical devices (e.g., a chest tube). Radiotherapy as an alternate treatment choice may be a more attractive option for those with a lower SES who may not be able to get time off work or fear a loss of income from a hospital stay lasting several days, or ongoing postoperative care. Interestingly, the association between neighbourhood income and use of radiotherapy appeared non-linear: patients in the highest income quintiles had greater odds of receiving radiotherapy compared with those in the lowest quintile, whereas no clear gradient was observed across intermediate quintiles. This pattern may reflect complex interactions between health status, treatment preferences, and the ability to absorb the logistical and financial costs of daily radiotherapy among higher-income patients who are not surgical candidates.

Geographic proximity to a regional cancer centre was associated with a higher likelihood of receiving surgery. After adjustment, patients living 50–100 km from a regional cancer centre had significantly lower odds of receiving surgery and higher odds of receiving radiotherapy compared with those living within 50 km, whereas no such differences were observed among patients living >100 km away. This non-linear pattern by distance may reflect that patients living 50–100 km from a regional centre face substantial travel and logistical barriers that make multi-day hospitalization and repeated pre- and post-operative visits less feasible, leading to greater reliance on radiotherapy. By contrast, patients living >100 km away may constitute a more highly selected group of fitter individuals who successfully navigate referral pathways to reach surgical centres, paralleling observations from broader literature [5,7] on travel distance and access to cancer treatment. Anticipating difficulties in patient adherence to surgical follow up and access to a specialist for possible postoperative complications may prompt referring physicians to choose radiotherapy over surgery. Despite a universal healthcare system in Ontario, geographical barriers to optimal treatment persist and remain an important area for ongoing research and policy change.

This is the first population-based study in Canada that has assessed the impact of SDOHs on type of treatment received for stage I lung cancer. The strengths of this study include a large cohort, a long study period, and an unselected cohort of patients across multiple institutions throughout a wide geographical area. There are limitations, however, inherent in the use of healthcare administrative databases. Firstly, only those with an Ontario health card are captured in these databases and registries. Secondly, despite our best attempts at controlling for possible confounding, some data may not have been captured, such as surgical contraindications. Third, we did not know if the immigrant group developed lung cancer before or after immigrating, which may have impacted how they accessed the healthcare system. We could not examine heterogeneity within the immigrant population according to immigration category, country of origin, or language proficiency, all of which may influence access to care and treatment decisions. Lastly, granular data such as a patient’s social situation (e.g., access to reliable public transport) may have impacted outcomes and could not be captured.

In summary, we have identified several SDOHs that affect treatment type for stage I lung cancer, including being rostered with a family physician, immigrant status, neighbourhood income quintile, and distance from a cancer centre. These disparities highlight the need for policy interventions aimed at improving equitable access to primary care, lung cancer screening, and surgical evaluation.

## Figures and Tables

**Table 1:** Distribution [n (row %)] of demographics and social determinants of health based on treatment received.

Variable	No Treatment	Surgery	Radiation	Total
**Age groups**				
<50	56 (11.6)	416 (86.5)	9 (1.9)	481
50–59	217 (11.5)	1524 (80.9)	143 (7.6)	1884
60–69	1015 (19.5)	3617 (69.4)	578 (11.1)	5210
70–79	1919 (27.6)	3816 (55.0)	1208 (17.4)	6943
≥80	2068 (49.1)	1059 (25.1)	1088 (25.8)	4215
**Sex**				
Male	2445 (30.4)	4188 (52.1)	1407 (17.5)	8040
Female	28,530 (26.5)	6244 (58.4)	1619 (15.1)	10,693
**Comorbidities**				
0	1246 (21.7)	3725 (65.0)	763 (13.3)	5734
1–2	1399 (32.9)	2032 (47.9)	815 (19.2)	4246
3–4	535 (43.0)	407 (32.7)	303 (24.3)	1245
≥5	165 (45.3)	99 (27.2)	100 (27.5)	364
**Frailty Index**				
No	4175 (25.2)	9851 (59.5)	2540 (15.3)	16,566
Yes	1100 (50.8)	581 (26.8)	486 (22.4)	2167
**Family Physician**				
Rostered	4368 (27.6)	8941 (56.4)	2542 (16.0)	15,851
Virtually rostered	716 (50.7)	301 (21.3)	394 (27.9)	1411
Not rostered	191 (40.6)	190 (40.3)	90 (19.1)	471
**Immigration**				
No	4976 (28.5)	9560 (54.7)	2927 (16.8)	17,463
Yes	299 (23.5)	872 (68.7)	99 (7.8)	1270
**Household Income**				
Quintile 1	472 (21.8)	1443 (66.7)	250 (11.5)	2165
Quintile 2	751 (25.1)	1826 (61.0)	418 (14.0)	2995
Quintile 3	976 (26.8)	2097 (57.6)	567 (15.6)	3640
Quintile 4	1187 (29.6)	2139 (53.3)	690 (17.2)	4016
Quintile 5	1829 (32.2)	2849 (50.2)	1002 (17.6)	5680
**Neighbourhood Income**				
Quintile 1	1579 (34.2)	2220 (48.1)	816 (17.7)	4615
Quintile 2	1190 (28.6)	2268 (54.5)	704 (16.9)	4162
Quintile 3	937 (26.5)	2038 (57.6)	561 (15.9)	3536
Quintile 4	826 (24.9)	2005 (60.5)	481 (14.5)	3312
Quintile 5	713 (23.5)	1870 (61.7)	448 (14.8)	3031
**Distance**				
<50 km	3800 (27.0)	8109 (57.7)	2142 (15.2)	14,051
50–100 km	1043 (31.5)	1641 (49.5)	632 (19.1)	3316
>100 km	412 (31.0)	670 (50.5)	246 (18.5)	1328
**Year**				
2007–2009	416 (15.8)	1711 (64.9)	510 (19.3)	2637
2010–2014	1098 (20.3)	3129 (57.8)	1186 (21.9)	5413
2015–2019	1885 (27.2)	3924 (56.6)	1124 (16.2)	6933
2020–2023	1876 (50.0)	1668 (44.5)	206 (5.5)	3750
**Region**				
Central	1185 (23.3)	3323 (65.3)	581 (11.4)	5089
Southwest	1520 (27.6)	3217 (58.3)	778 (14.1)	5515
Eastern	1818 (32.7)	2528 (45.5)	1213 (21.8)	5559
Northern	736 (28.9)	1358 (53.3)	452 (17.8)	2546
**New Immigrant**				
No	5234 (28.2)	10,335 (55.6)	3018 (16.2)	18,587
Yes	41 (28.1)	97 (66.4)	8 (5.5)	146

Note: The table does not include patients that received surgery + radiation (N = 446) but they are included in the multivariable analysis.

**Table 2:** Variables associated with receiving surgery.

	OR	95%CI	*p*-Value
**Age groups**			
<50 (reference)	---	---	---
50–59	0.66	0.45–0.96	0.032
60–69	0.44	0.31–0.62	<0.001
70–79	0.26	0.18–0.37	<0.001
≥80	0.07	0.05–0.10	<0.001
**Sex**			
Male (reference)	---	---	---
Female	1.17	1.07–1.27	<0.001
**Comorbidities**			
0 (reference)	---	---	---
1–2	0.5	0.45–0.54	<0.001
3–4	0.31	0.27–0.36	<0.001
≥5	0.21	0.16–0.27	<0.001
**Frailty Index**			
No (reference)	---	---	---
Yes	0.38	0.34–0.44	<0.001
**Family Physician**			
Rostered (reference)	---	---	---
Virtually rostered	0.85	0.75–0.96	0.009
Not rostered	0.59	0.44–0.78	<0.001
**Immigration**			
No (reference)	---	---	---
Yes	1.23	1.01–1.50	0.035
**Household Income**			
Quintile 1 (reference)	---	---	---
Quintile 2	0.86	0.72–1.01	0.07
Quintile 3	0.87	0.74–1.03	0.109
Quintile 4	0.77	0.65–0.92	0.003
Quintile 5	0.69	0.58–0.82	<0.001
**Neighbourhood Income**			
Quintile 1 (reference)	---	---	---
Quintile 2	1.32	1.16–1.51	<0.001
Quintile 3	1.35	1.17–1.55	<0.001
Quintile 4	1.35	1.16–1.58	<0.001
Quintile 5	1.45	1.24–1.71	<0.001
**Distance**			
<50 km (reference)	---	---	---
50–100 km	0.84	0.75–0.95	0.004
>100 km	0.93	0.78–1.12	0.449
**Year**			
2007–2009 (reference)	---	---	---
2010–2014	0.65	0.57–0.74	<0.001
2015–2019	0.55	0.48–0.62	<0.001
2020–2023	0.33	0.28–0.38	<0.001
**Region**			
Central (reference)	---	---	---
Southwest	0.78	0.69–0.88	<0.001
Eastern	0.36	0.32–0.41	<0.001
Northern	0.57	0.49–0.67	<0.001

**Table 3:** Variables associated with receiving radiation.

	OR	95%CI	*p*-Value
**Age groups**			
<50 (reference)	---	---	---
50–59	2.95	1.56–5.57	0.001
60–69	4.56	2.47–8.43	<0.001
70–79	6.3	3.42–11.61	<0.001
≥80	9.86	5.34–18.21	<0.001
**Sex**			
Male (reference)	---	---	---
Female	0.85	0.77–0.93	0.001
**Comorbidities**			
0 (reference)	---	---	---
1–2	1.46	1.32–1.63	<0.001
3–4	1.67	1.43–1.94	<0.001
≥5	2.24	1.75–2.86	<0.001
**Immigration**			
No (reference)	---	---	---
Yes	0.69	0.53–0.89	0.005
**Household Income**			
Quintile 1 (reference)	---	---	---
Quintile 2	1.16	0.95–1.41	0.151
Quintile 3	1.09	0.90–1.32	0.39
Quintile 4	1.21	1.00–1.46	0.051
Quintile 5	1.27	1.06–1.52	0.009
**Year**			
2007–2009 (reference)	---	---	---
2010–2014	1.12	0.98–1.29	0.102
2015–2019	0.81	0.71–0.93	0.003
2020–2023	0.24	0.20–0.30	<0.001
**Region**			
Central (reference)	---	---	---
Southwest	1.26	1.10–1.45	0.001
Eastern	2.11	1.84–2.41	<0.001
Northern	1.65	1.40–1.94	<0.001

## Data Availability

The original contributions presented in this study are included in the article. Further inquiries can be directed to the corresponding author.
